# iHear: Canadian medical student based hearing assessment program for grade school children using a tablet audiometer

**DOI:** 10.1186/s40463-021-00542-w

**Published:** 2021-10-29

**Authors:** Deanna Lammers, Adam Rocker, David S. Chan, Deema Couchman, Yiqiao Wang, Amy Fraser, Johnna MacCormick, Matthew Bromwich

**Affiliations:** 1 Division of Otolaryngology - Head and Neck Surgery, Dalhousie University, Halifax, Canada; 2 Faculty of Medicine, University of Alberta, Edmonton, Canada; 3 Department of Otolaryngology - Head and Neck Surgery, McGill University, Montreal, Canada; 4 Faculty of Medicine, University of Toronto, Toronto, Canada; 5 Children’s Hospital of Eastern Ontario, 401 Smyth Rd, Ottawa, ON K1H 8L1 Canada

**Keywords:** Hearing loss, Tablet audiometry, Mobile technology, Medical education, Pediatric healthcare

## Abstract

**Purpose:**

To evaluate the progress and challenges of a hearing screening program as well as review the incidence of pediatric hearing loss in grade school children participating in this program.

**Methods:**

Medical students from the University of Ottawa established iHear, a grade school hearing assessment program that uses novel tablet audiometry. Over 3 years, children in grades 1 and 2 were assessed and those found to have abnormal results on iHear assessment were then referred to audiology for formal testing, and to otolaryngology if needed.

**Results:**

From 2014 to 2017, 753 children aged 5–9 years old were assessed for hearing loss. Mean age of participants was 6.7 years, 51.9% of whom were female. Of the children assessed, 86 (11.4%) had abnormal results and 6 (0.8%) had inconsistent results, necessitating 92 referrals for assessment by a professional audiologist. Of the 65 participants who completed secondary audiologic assessment, 54 (83.1%) were normal and 11 (16.9%) had a definitive hearing loss or abnormal tympanometry. A total of 32 children were lost to follow-up. A total of 118 medical students were involved in the iHear program.

**Conclusions:**

Hearing loss in grade school populations continues to go undetected across Canada. Programs such as iHear demonstrate that gaps in the provision of hearing assessment can be filled effectively by medical students equipped with tablet audiometry. Medical student exposure to audiology and otolaryngology increased through the iHear program.

**Graphical Abstract:**

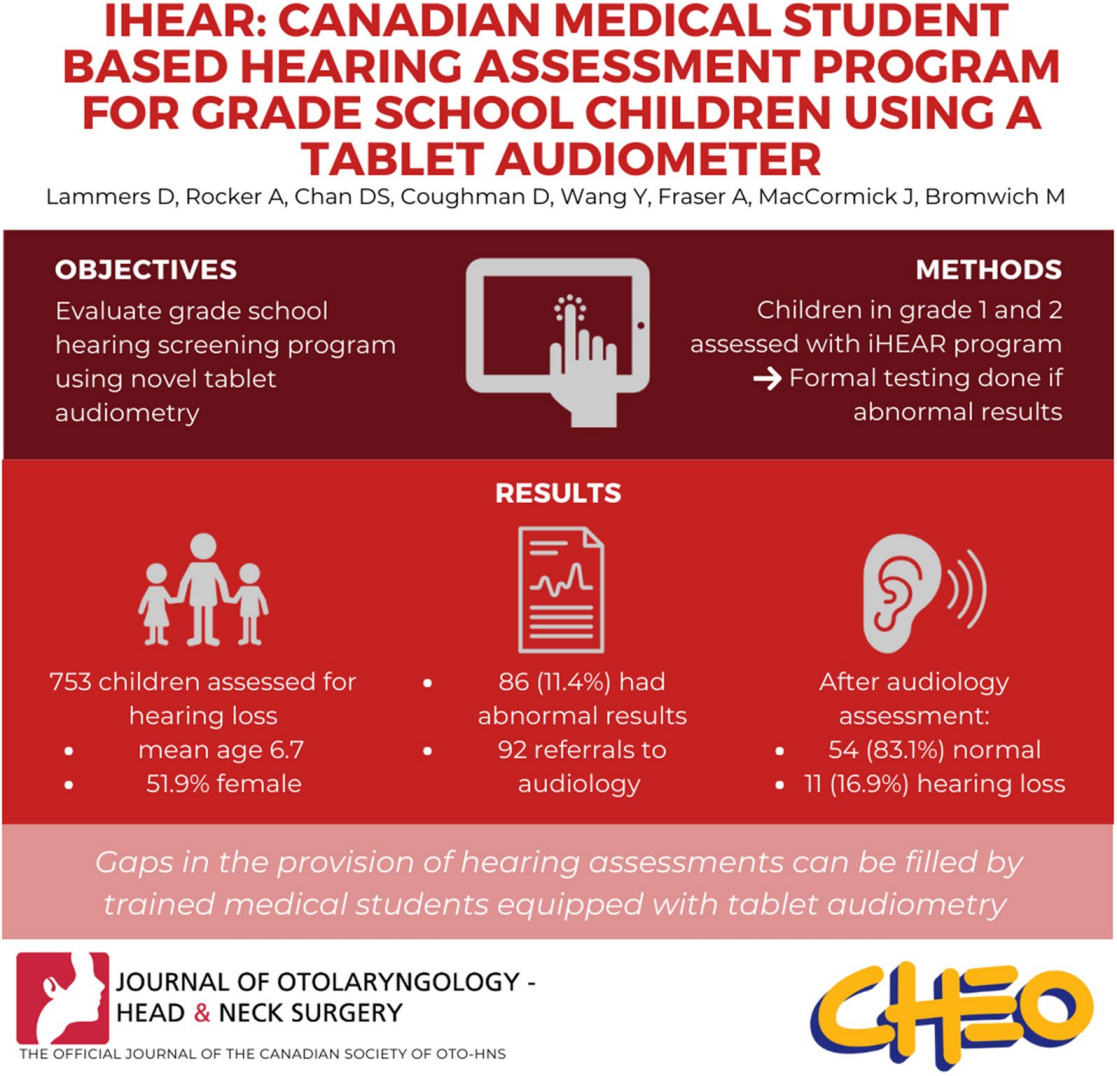

## Introduction

Over 34 million children are affected by disabling hearing loss (HL) worldwide [1]. Without proper detection of HL, children may experience delays in language acquisition, impaired intellectual and social development, increased rates of mental illness, and downstream under- and un-employment [1–6]. Early identification of HL, along with timely intervention, proper treatment, and ongoing audiology support can prevent these negative outcomes [3, 7]. Interventions may include the fitting of hearing aids or cochlear implants as well as services such as speech therapy and aural rehabilitation [1].

The American Academy of Paediatrics recommends that “objective screenings for hearing impairment should be performed periodically on all infants and children” [8]. Despite this, no HL screening program exists for school-aged children in Canada [5].

Historically, childhood HL screening programs have been limited by access to audiology services [5]. Soundproof booths, desktop audiometers, and professionally trained audiologists are the gold standard for audiometric assessment but come at a cost. SHOEBOX® Audiometry (SHOEBOX Ltd., 2018) is a tablet audiometry application that has demonstrated 93.3% sensitivity and 94.5% specificity for hearing loss when compared with conventional desktop audiometry in a sound booth [9]. It has also demonstrated robust utility in providing hearing assessments to Canada’s healthcare resource-limited North [10].

To address the lack of grade school hearing assessments in Ontario, medical students from the University of Ottawa have developed iHear, a grade school hearing assessment program. The program uses tablet audiometry to screen children in grades 1 and 2 for hearing impairment in the Ottawa region [11]. Children flagged for possible HL are referred to audiology for a more detailed assessment, and subsequently to otolaryngology as needed. The iHear program documents outcomes at all stages of this process to better define rates of HL in the Ottawa grade school population and to quantify referral effectiveness.

This paper aims to evaluate the iHear hearing assessment program and to report on the incidence of paediatric HL in grade school children participating in this program.

## Methods

### Ethics approval

This study was approved in full by the Children’s Hospital of Eastern Ontario Research Ethics Board (protocol 14/170X), the University of Ottawa’s Office of Research Ethics and Integrity (protocol A02-15–01), the Ottawa-Carleton Research and Evaluation Advisory Committee, and involved schoolboards.

### iHear medical student recruitment and training

iHear technicians were recruited annually from 1st and 2nd year cohorts of the University of Ottawa Medical Undergraduate program. Prior to participation on any school visit, the medical students were required to attend a full 2-h training session in which they were familiarized with consequences of HL, hearing anatomy, causes of HL, otoscopy, and audiometry, and given practical training with otoscopes and the tablet audiometer. Alternatively, students could complete AE100: Audiometry Essentials, an online training module developed to provide a foundational understanding of audiometry and hearing assessment, and then attend a 1-h practical training session with otoscopes and tablet audiometers. Training was provided by an Otolaryngologist as well as medical student iHear executive members who had extensive involvement in the program and additional training.

### Hearing assessment and follow-up

**Fig. 1:**
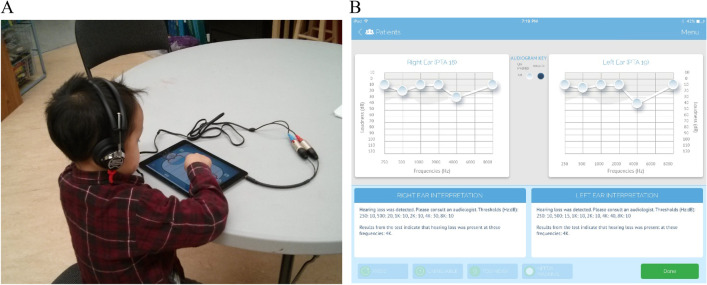
SHOEBOX tablet audiometer application on a tablet computer paired with TDH-50 audiometry headphones (**A**) and in-application audiogram displaying bilateral HL at 4000 Hz (**B**)

Schools in the Ottawa region were contacted annually to arrange school visits. During each visit, children in grades 1 and 2 underwent a hearing assessment using SHOEBOX® Audiometry, an iPad-based mobile audiometer (Fig. 1). Automated pure tone air conduction audiometry was performed with the audiometer application and TDH-50 audiometry headphones. Children were defined as having an abnormal result if any of the hearing thresholds obtained at 1000, 2000, and 4000 Hz were ≥ 20 dB HL, or ≥ 30 dB HL at 500 Hz in one or both ears. Children who were determined to have an abnormal or a persistently inconclusive result based on the tablet audiometry were referred to the University of Ottawa Interprofessional Rehabilitation Clinic for conventional audiometry including bone conduction audiometry, tympanometry, and otoacoustic emission testing. If an abnormality was present at conventional audiometric assessment, children were referred to an Otolaryngologist for further medical follow-up.

### Statistical analysis

Data analysis was performed using Microsoft Excel 2016 software (Microsoft Office 365 ProPlus, Version 1708, Build 8431.2250). Graphs were produced using GraphPad Prism software (GraphPad Software, Version 6.01).

## Results

**Fig. 2:**
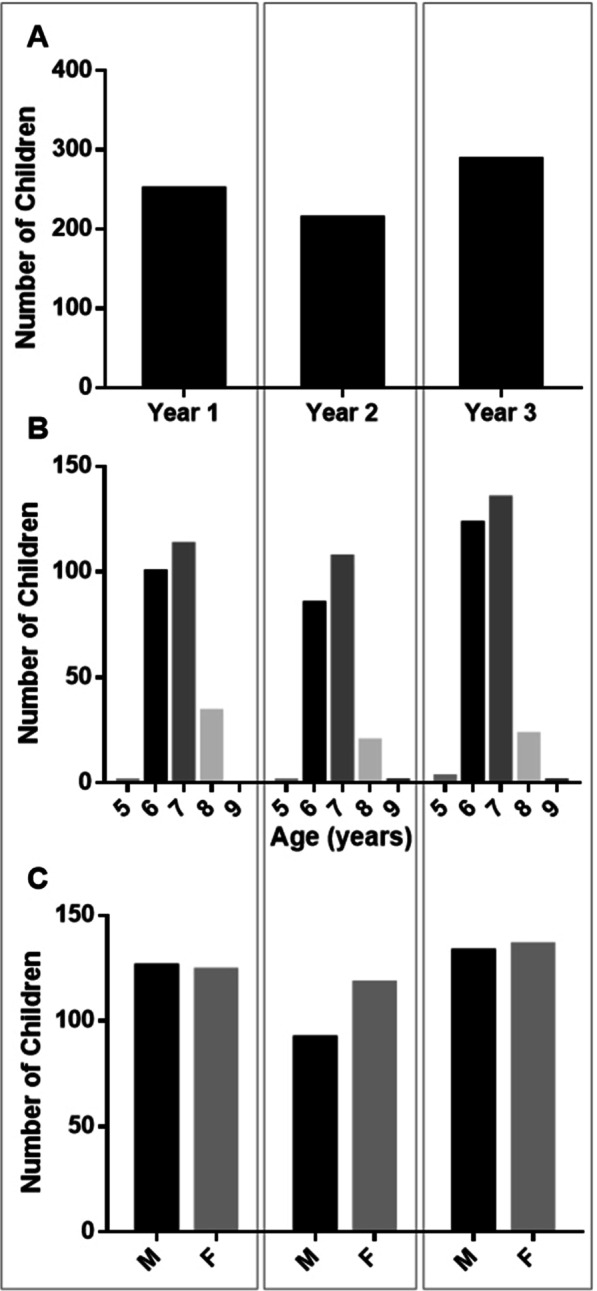
Annual iHear program demographics. Number (**A**), age (**B**), and gender (**C**) of children assessed in each respective year of the program. M, male, F, female

Between September 2014 and May 2017, the Ottawa iHear program recruited and assessed 753 grade school children for HL in the Ottawa region (Fig. 2). Participants ranged in age from 5 to 9 years old. The majority (88.8%) of participants were 6 or 7 years old with a mean age of 6.7 years at time of assessment. Female participants made up 51.9% of the study population.

**Fig. 3:**
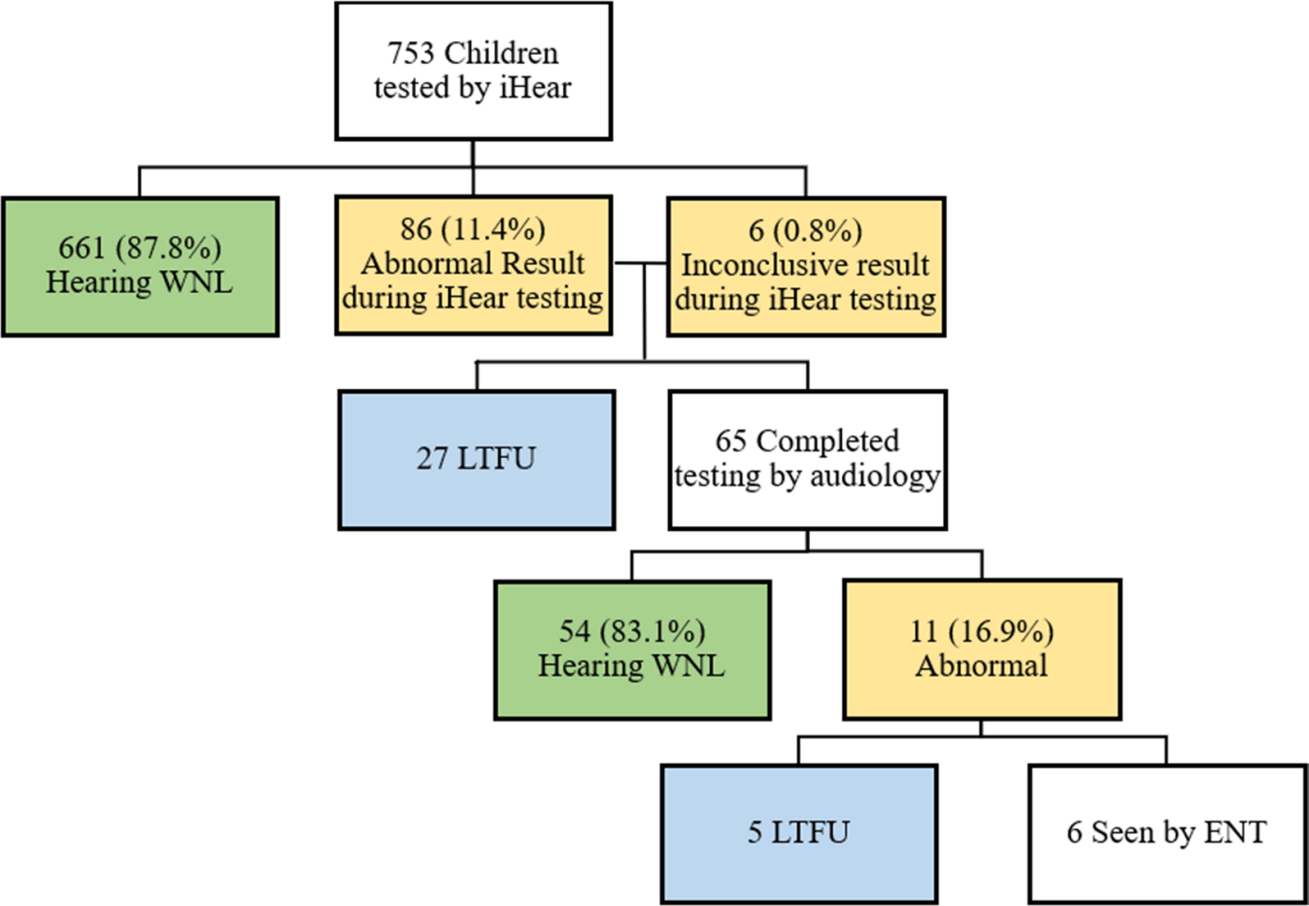
iHear assessment outcomes. WNL, within normal limits; LTFU, lost to follow-up; ENT, otolaryngology

Of the 753 children assessed as part of the iHear program, 86 (11.4%) had abnormal results and 6 (0.8%) had inconsistent results, necessitating 92 referrals for assessment by a professional audiologist (Fig. 3). Of the referred participants, 27 were lost to follow-up (LTFU) and 65 completed audiologic assessment at the University of Ottawa Interprofessional Rehabilitation Clinic or the Children’s Hospital of Eastern Ontario. Mean time to audiologic assessment from iHear assessment was 69.2 ± 77.6 days (Table 1). Upon conclusion of audiologic assessment, 54 (83.1%) were normal and 11 (16.9%) had a definitive HL or abnormal tympanometry. As such, the abnormal hearing positive predictive value of iHear assessment is 16.9%. Of the 11 cases of abnormal outcome on audiologic assessment, 8 were conductive HL, 2 were sensorineural HL, and 1 was isolated abnormal tympanometry. As per protocol, all 11 abnormal cases were referred for further assessment by an Otolaryngologist. Of those referred to otolaryngology, 5 were LTFU and 6 were assessed by an Otolaryngologist at the Children’s Hospital of Eastern Ontario. Mean time to otolaryngology from iHear assessment was 299.8 ± 162.8 days. In total, 32 children were LTFU. The most common reason for a child to be LTFU was due to lack of reliable contact information (no response when called, incorrect phone number, discontinued phone number). Additional reasons included refusal of services by a parent/guardian, language barrier, and follow-up elsewhere such that results were not accessible by the research team.

In the first 3 years of the iHear program, 9 training sessions were held and 118 medical students were trained as iHear technicians (Table 2). Thirteen schools were involved in the program and 46 visits took place. On average, 16.4 children were assessed per visit.

## Discussion

Of the 753 children assessed by iHear, 11.4% had abnormal results (Fig. 3). This is greater than the 5.4% prevalence of HL in Canadian children aged 6–11 reported by Feder et al. [12]. The observed discrepancy in HL prevalence could be related to a number of factors in the present work such as inclusion of children with otoscopic abnormalities (ear infection, excess cerumen), administration of assessment by 1st and 2nd year medical students versus health measures specialists trained by a certified audiologist, difference in age range (5 to 9 versus 6 to 11), and geographic differences in HL prevalence between Ottawa and the rest of Canada. Furthermore, in the current work, the threshold for background noise was exceeded on a few visits due to lack of quiet room availability on school property. The shortage of quiet rooms may have affected the quality of the program [13].

Of those referred to audiology for evaluation, 11 of the 65 who completed assessment had a definitive HL (Fig. 3). As such, the iHear program has a positive predictive value for abnormal hearing of 16.9%. Middle ear pathologies associated with conductive HL such as acute otitis media or otitis media with effusion are common in the assessed age group and resolve spontaneously in most cases within 3 weeks or 3 months, respectively [14–16]. Given that the mean time between iHear and audiologic assessment was 69.2 days (Table 1), it is difficult to make conclusions on the positive predictive value and the true value of iHear assessment is likely higher [10]. While no comparable school-age HL screening program currently exists in Canada [5], in 2008/2009 the Gift of Sight and Sound program allocated resources to perform mobile audiometry, tympanometry, and otoacoustic emission testing at 6 schools in the Toronto area. Assessment was performed by international physicians and health care professionals from the Canadian Hearing Society. The program referred 27 children on for audiologic assessment and had a positive predictive value of 20.7% [5]. It is encouraging that the positive predictive value of the iHear program was similar given the singular use of novel tablet play audiometry performed by medical students.

Following iHear referral, consultation with otolaryngology was completed in 6 cases with a mean time between iHear and otolaryngology assessment of 299.8 days (Table 1). Accounting for the mean time to audiology of 68.2 days, this is still a wait of almost 8 months to see a specialist regarding hearing outcomes and is in keeping with Health Quality Ontario data that shows 56% of pediatric otolaryngology patients are assessed within target time at the Children’s Hospital for Eastern Ontario [17]. This emphasizes a need for increased access to pediatric otolaryngology services.

Developed from the ground up by medical student volunteers, the iHear program has achieved great popularity at the University of Ottawa Faculty of Medicine. Over the first 3 years of the program 118 medical students have been involved as iHear technicians (Table 2). This volunteer base represents roughly one quarter of the University of Ottawa undergraduate medical student population. Mean volunteer commitment was 7.73 h (data not shown), including at least 2 h of training in audiometry, with the balance spent at schools performing hearing assessments. According to the University of Ottawa Undergraduate Medical Education curriculum, students receive a cumulative 7 h of lectures and practical exposure to hearing and audiology. Thus, through recruitment of medical students to the iHear program, exposure to audiometry is effectively doubled.

The iHear program exists through collaboration with the University of Ottawa Interprofessional Rehabilitation Clinic and Otolaryngologists at the Children’s Hospital of Eastern Ontario. Unfortunately, despite the best efforts of these organizations and the iHear executives, one of the largest issues faced by the program was the number of children LTFU. The most common reason for a child to be LTFU was due to lack of reliable contact information. Similar issues with follow-up were experienced by the Toronto-based Gift of Sight and Sound program, with specific barriers identified by interviewing participant’s parents: Lack of medical insurance coverage, lack of accessible transportation, inability for parents to take time off from work, and lack of confidence in navigating the health care system due to cultural and language barriers [5]. To decrease rates of LTFU, additional contact information fields have been added to consent forms and the follow-up process has been optimized through collaboration with the University of Ottawa Interprofessional Rehabilitation Clinic. Future optimization could include identification of the primary language spoken at home to aid follow-up planning as well as correlate with assessment outcomes.

In summary, the Ottawa iHear program has employed novel mobile audiometry technology in assessing 753 children over 3 years, identifying otherwise undetected cases of HL and connecting affected individuals with the healthcare resources they require. While the assessed cohort represents only a small proportion of children within the Ottawa area, it reflects a much larger need for grade school hearing assessments locally, provincially, and nationally. To that end, the iHear program has expanded to medical school campuses at the University of Saskatchewan, McGill University, and Dalhousie University. While expansion of the iHear program will provide access to hearing assessments for more children across Canada, the program as it stands is inherently limited to cities associated with medical school campuses.

## Conclusion

In conclusion, hearing loss in grade school populations continues to go undetected across Canada. Programs such as iHear demonstrate that gaps in hearing assessment service provision can be filled effectively by local technicians equipped with novel tablet play audiometry.

**Table 1:** Time from iHear testing to audiology and otolaryngology assessment

Mean time from iHear assessment	Time ± standard deviation (days)
To audiology	69.2 ± 77.6
To otolaryngology	299.8 ± 162.8

**Table 2:** Annual iHear Ottawa program metrics

Program metric	Year 1	Year 2	Year 3	Total
Training sessions held	4	2	3	9
Medical students trained	40	38	40	118
Participating schools	8	4	6	13^a^
School visits	14	13	19	46
Children assessed per visit	17.9	16.5	15.2	16.4

^a^Represents the total number of unique schools visited by iHear, as some schools were visited in multiple years

## Data Availability

All data generated or analysed during this study are included in this published article.

## Abbreviations

HL: Hearing loss
LTFU: Lost to follow-up
M: Male
F: Female
WNL: Within normal limits
ENT: Otolaryngology
